# Pemphigoid gestationis: a rare pregnancy-associated autoimmune blistering disorder

**DOI:** 10.1007/s00404-025-08146-9

**Published:** 2025-08-19

**Authors:** Sivan Farladansky-Gershnabel, Gil Shechter-Maor, Tal Biron-Shental

**Affiliations:** 1 Department of Obstetrics and Gynecology, Meir Medical Center, 44281 Kfar Saba, Israel; 2 Gray Faculty of Medical & Health Sciences, Tel Aviv University, Tel Aviv, Israel

## Case Presentation

**Fig. 1:**
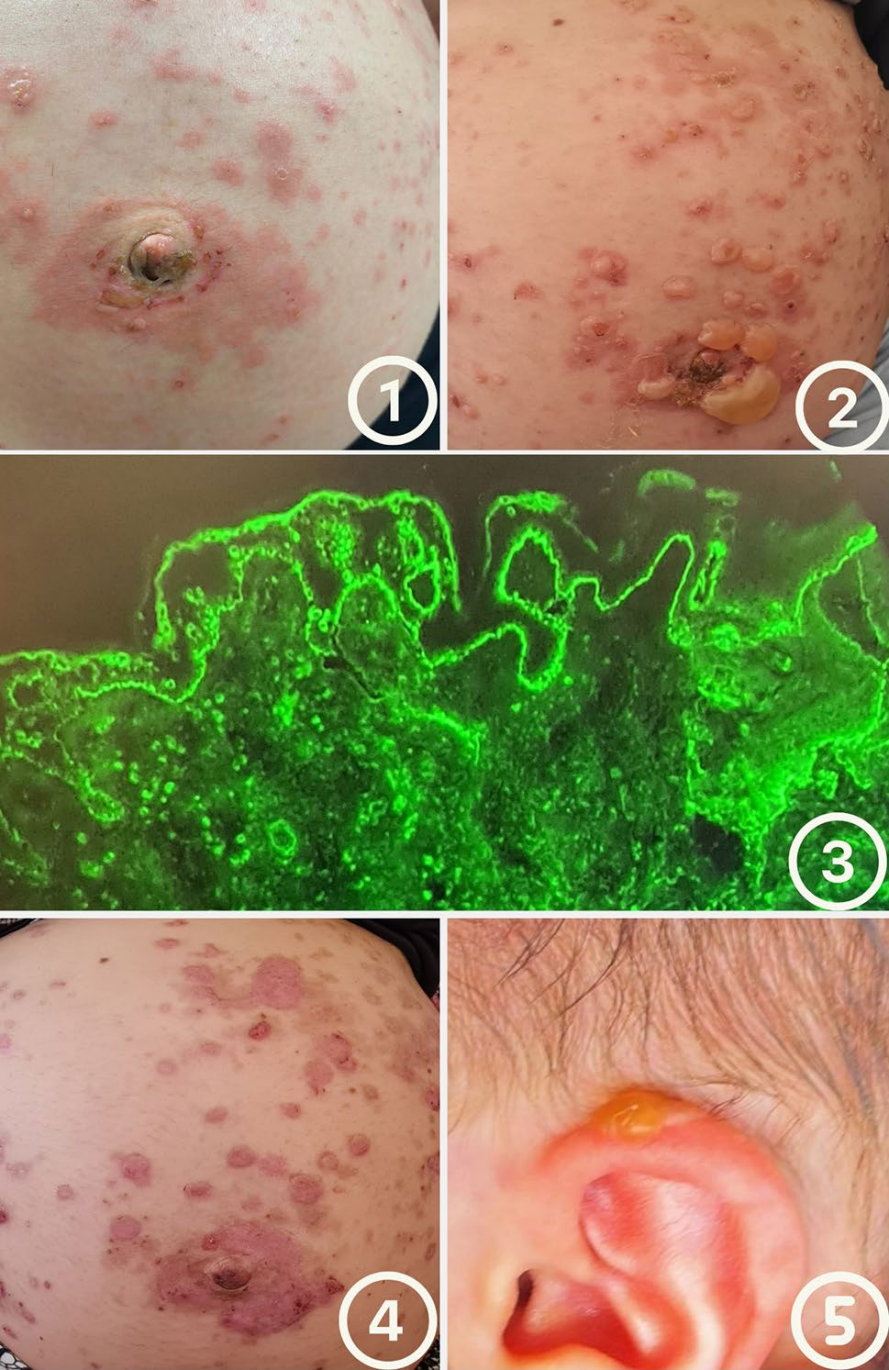
Clinical and immunopathologic findings in pemphigoid gestationis. **1** Initial presentation with periumbilical erythematous plaques and vesicles. **2** Progression to tense bullae with surrounding erythema. **3** Direct immunofluorescence of perilesional skin showing linear deposition of C3 and IgG along the basement membrane zone. **4** Later stage with residual hyperpigmentation and resolving plaques. **5** Neonatal involvement with an isolated bullous lesion on the auricle, which resolved spontaneously

A 28-year-old gravida 2, para 1 (G2P1) woman at 33 weeks of gestation presented to the emergency department with a pruritic periumbilical rash composed of confluent erythematous patches and sparse vesicles (Fig. 1). The patient denied systemic symptoms, medication use, recent infections, or allergies. She also denied recent travel, fever, or myalgia. Her current pregnancy had been uneventful, and her previous delivery was a full-term spontaneous vaginal birth.

Initial examination revealed erythematous plaques measuring 3–5 cm, involving approximately 30–40% of the body surface area, predominantly over the trunk and abdomen. Within days, these lesions evolved into large, tense bullae affecting the abdomen, trunk, and back (Fig. 1.2). Vital signs were within normal limits. There were no signs of hepatosplenomegaly. Fetal well-being was confirmed via ultrasound and non-stress testing, demonstrating normal fetal growth and activity.

Laboratory evaluation showed normal white blood cell and eosinophil counts, liver enzymes, and C-reactive protein levels. Blister fluid was PCR-negative for herpes simplex virus and varicella-zoster virus.

Histopathologic examination of lesional skin demonstrated a subepidermal blister with a mixed inflammatory infiltrate rich in eosinophils. Direct immunofluorescence (DIF) of perilesional skin revealed linear deposition of C3 and IgG along the basement membrane zone, consistent with a diagnosis of pemphigoid gestationis (Fig. 1.3).

She was admitted to the fetal-maternal unit for monitoring and treatment. She was managed with systemic corticosteroids, starting at 60 mg/day of prednisone, along with oral antihistamines and topical antibiotic cream. Over a week, the blisters gradually crusted and became less itchy (Fig. 1.4).

At 38 weeks, the patient delivered a healthy male infant weighing 2905 g with Apgar scores of 9 and 10 at 1 and 5 min, respectively. On the second day of life, the neonate developed one small blister on the left auricle and another on the chin (Fig. 1.5), both of which resolved spontaneously. No new lesions appeared prior to discharge, and physical examination and vital signs remained normal throughout hospitalization.

## Discussion

Pemphigoid gestationis (PG) is a rare autoimmune bullous dermatosis of pregnancy, with an estimated incidence of 1 in 40,000 to 50,000 pregnancies [1, 2]. The disease is mediated by maternal IgG autoantibodies directed against the NC16a domain of BP180 (collagen XVII), a hemidesmosomal transmembrane protein at the dermo-epidermal junction [3, 4]. Antibody binding initiates complement activation, especially deposition of C3, leading to eosinophilic infiltration and subepidermal blister formation [5].

Clinically, PG usually presents in the second or third trimester with a periumbilical pruritic urticarial rash that progresses to tense vesicles and bullae, often spreading to the trunk and extremities [2, 6]. Mucosal involvement is rare.

Importantly, maternal autoantibodies of the IgG1 subclass can cross the placenta, especially during the third trimester, and bind to the fetal skin basement membrane zone. As a result, approximately 5–10% of neonates born to affected mothers may exhibit transient bullous or urticarial lesions [3, 7]. These neonatal lesions typically appear within the first few days of life and resolve spontaneously over 1–2 weeks without scarring or systemic involvement. In our case, the neonate presented with two isolated bullae that resolved without intervention, consistent with this benign, self-limited course.

Beyond cutaneous involvement, PG has been associated with a slightly increased risk of adverse pregnancy outcomes. Several studies have reported higher rates of small for gestational age (SGA) neonates, preterm birth, and placental insufficiency in affected pregnancies [3, 4]. The pathophysiological basis for these outcomes is not fully understood, but may relate to placental deposition of immune complexes, complement activation, or generalized maternal inflammation. While most pregnancies proceed uneventfully with appropriate management, close fetal surveillance is recommended to detect early signs of growth restriction or compromise.

The differential diagnosis includes other pregnancy-associated dermatoses, such as polymorphic eruption of pregnancy (PEP), atopic eruption of pregnancy (AEP), and infectious or autoimmune blistering conditions. PEP typically arises in striae distensae during late pregnancy and lacks blistering. AEP usually occurs earlier and is associated with eczematous papular lesions and a history of atopy [8]. Bullous impetigo can present with vesicles but is caused by Staphylococcus aureus and is confirmed by bacterial culture, with negative DIF findings.

Diagnosis of PG relies on clinical suspicion and is confirmed histologically by subepidermal blisters with eosinophilic infiltrates and immunopathologically by linear deposition of C3 and often IgG at the dermo-epidermal junction [1, 6]. These findings help distinguish PG from other bullous diseases of pregnancy and from pemphigus vulgaris or bullous pemphigoid, which have different immunopathologic patterns.

The mainstay of treatment is corticosteroids. Mild cases may respond to topical agents and antihistamines, but systemic corticosteroids (e.g., prednisone 0.5–1 mg/kg/day) are often necessary for moderate to severe disease [4, 9, 10] Disease activity can fluctuate and may worsen near term or postpartum. Recurrence is common in subsequent pregnancies or with hormonal triggers, such as menstruation or oral contraceptives [1, 4].

This case illustrates the clinical course and multidisciplinary management of pemphigoid gestationis, supported by histopathologic and immunofluorescence confirmation. The progression of maternal and neonatal lesions was meticulously documented through sequential clinical images, providing a rare and visually compelling representation of the disease. Such detailed photographic documentation enhances the educational value of this report and contributes to the limited visual literature on PG in both mother and newborn.

## Conclusion

Pemphigoid gestationis is a rare but potentially impactful autoimmune condition that requires timely diagnosis and careful multidisciplinary management. This case underscores the importance of clinical suspicion, confirmatory testing, and individualized treatment in optimizing outcomes for both mother and fetus. The thorough photographic documentation of lesion evolution in this report provides an important visual contribution to the literature and may assist clinicians in recognizing and managing similar cases in the future.

## Data Availability

No datasets were generated or analysed during the current study.
